# Supplementary material for: Prediction of final pathology depending on preoperative myometrial invasion and grade assessment in low-risk endometrial cancer patients: A Korean Gynecologic Oncology Group ancillary study
Source: PLoS One. 2024 Jun 27;19(6):e0305360. doi: 10.1371/journal.pone.0305360 (PMC11210801; doi:10.1371/journal.pone.0305360)
Supplement: S1 Fig — Logistic Regression was used to predict Group 1. Reference represents conventional analysis. * denotes the matching rate of the depth of MI between preoperative assessment and postoperative pathology. ** indicates the matching rates that were randomly increased. (PDF) [file pone.0305360.s001.pdf]

S1 Fig. Comparison of the AUC performance based on the increased matching rate between preoperative and postoperative depth of MI.

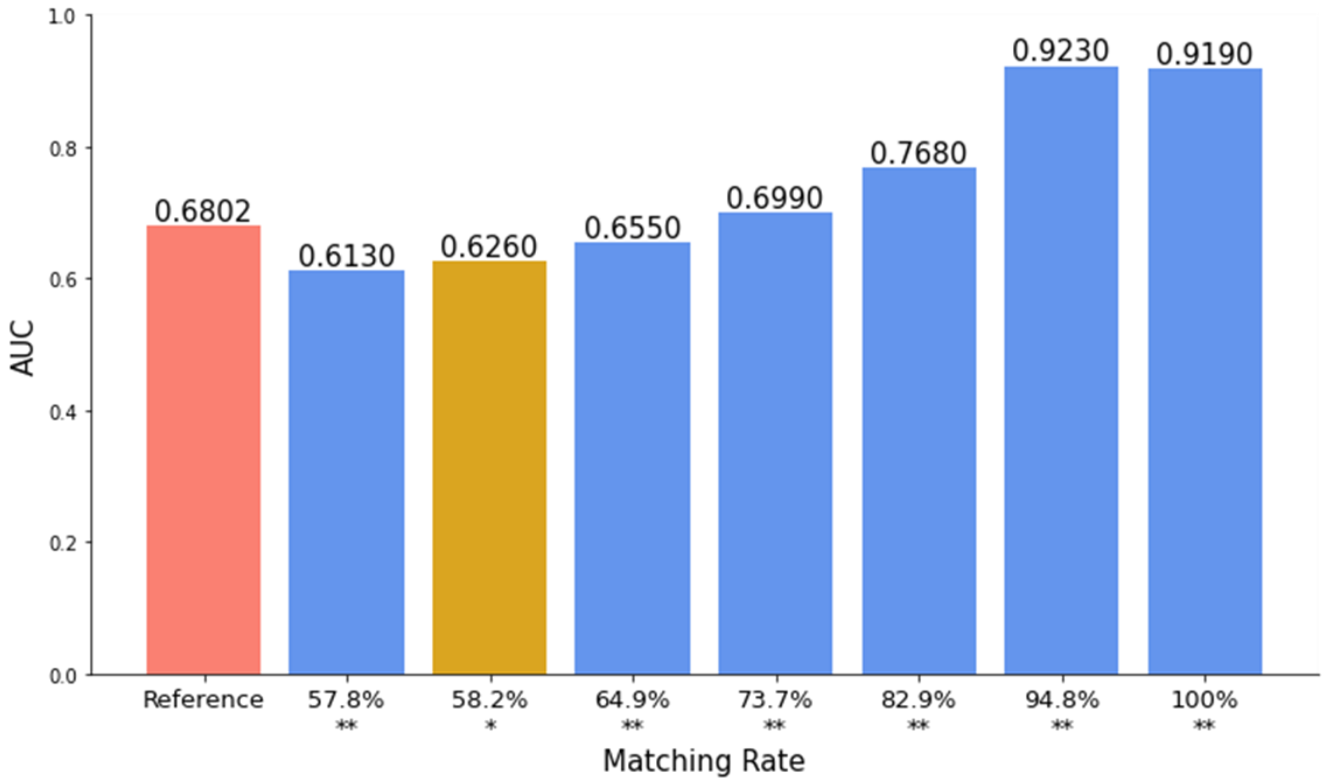

Logistic Regression was used to predict Group 1. Reference represents conventional analysis.

* denotes the matching rate of the depth of MI between preoperative assessment and postoperative pathology.

** indicates the matching rates that were randomly increased.

6
