# Supplementary material for: Prediction of final pathology depending on preoperative myometrial invasion and grade assessment in low-risk endometrial cancer patients: A Korean Gynecologic Oncology Group ancillary study
Source: PLoS One. 2024 Jun 27;19(6):e0305360. doi: 10.1371/journal.pone.0305360 (PMC11210801; doi:10.1371/journal.pone.0305360)
Supplement: S2 Fig — a. NPM1, b. NPM2. Value 0 means no MI, value 1 means MI <1/2, and value 2 means MI ≥1/2. MI, myometrial invasion; NPM 1, New Prediction Model 1; NPM 2, New Prediction Model 2. (PDF) [file pone.0305360.s002.pdf]

S2 Fig. Comparisons of the imputed depth of MI and postoperative depth of MI.

a

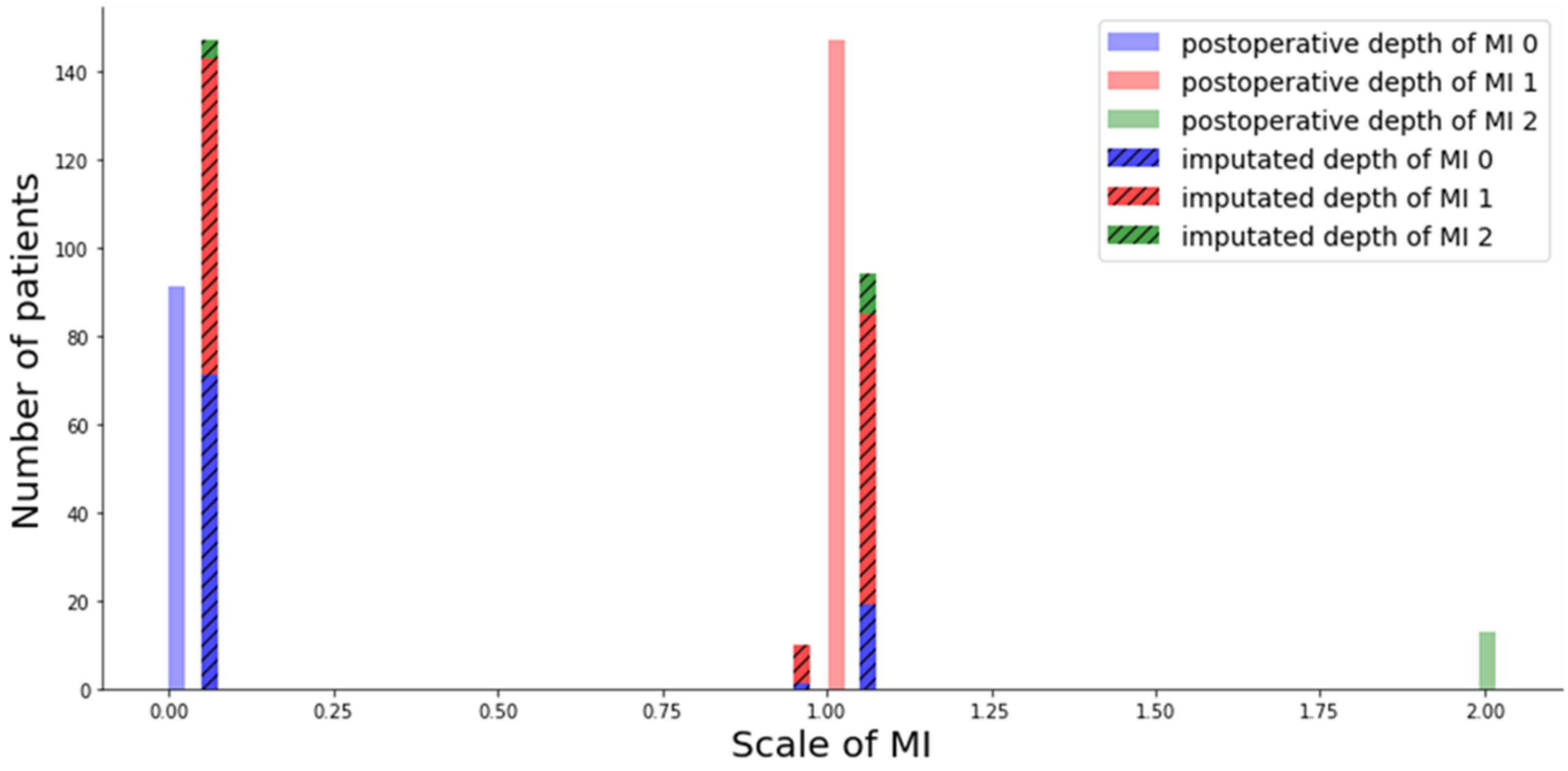

b

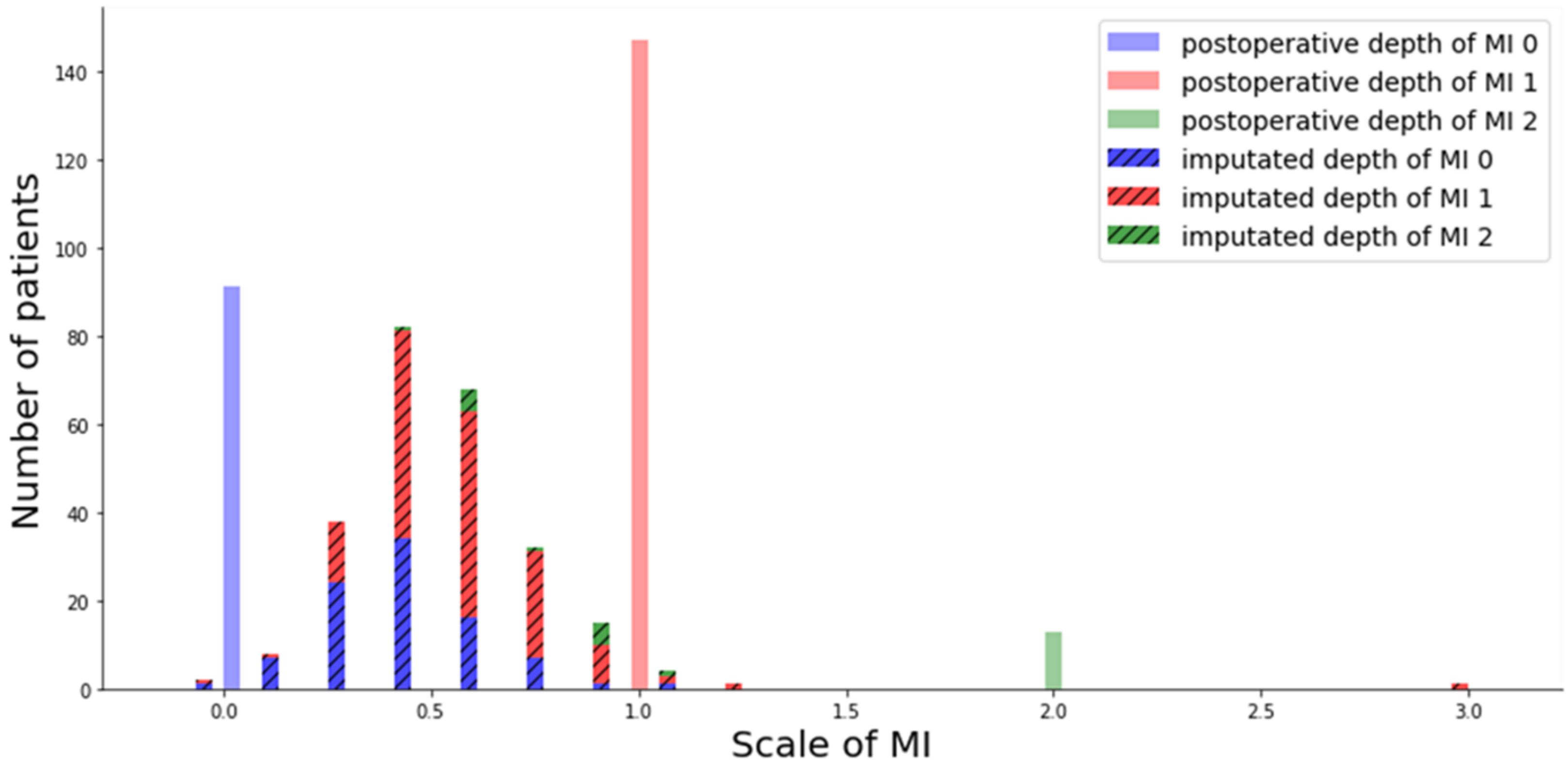

a. NPM1, b. NPM2. Value 0 means no MI, value 1 means MI <1/2, and value 2 means MI ≥1/2. MI, myometrial invasion; NPM 1, New Prediction Model 1; NPM 2, New Prediction Model 2.
