# Supplementary material for: Prediction of final pathology depending on preoperative myometrial invasion and grade assessment in low-risk endometrial cancer patients: A Korean Gynecologic Oncology Group ancillary study
Source: PLoS One. 2024 Jun 27;19(6):e0305360. doi: 10.1371/journal.pone.0305360 (PMC11210801; doi:10.1371/journal.pone.0305360)
Supplement: S1 Table — (PDF) [file pone.0305360.s003.pdf]

S1 Table. Relationship between the preoperative assessment and postoperative pathology in a higher-risk population than a low-risk population based on the postoperative final pathology.

	Preoperative classification											
	Total (n = 35/251)				Group 1 (n = 11/106)		Group 2 (n = 10/41)		Group 3 (n = 6/74)		Group 4 (n = 8/30)	
	MI		Grade		MI	Grade	MI	Grade	MI	Grade	MI	Grade
	No (n = 21)	<1/2 (n = 14)	1 (n = 17)	2 (n = 18)	No	1	No	2	<1/2	1	<1/2	2
**Postoperative final pathology**												
**Stage, n**												
**IA (grade 3)**	**11 (4.4%)**				**5**		**5**				**1**	
**IB**	**12 (4.8%)**				**3**				**5**		**4**	
**II**	**6 (2.4%)**						**2**		**1**		**3**	
**IIIC**	**6 (2.4%)**				**3**		**3**					
**MI, n**												
**No**	**9**	**1**			**5**		**4**		**-**		**1**	
**<1/2**	**8**	**4**			**3**		**5**		**1**		**3**	
**≥1/2**	**4**	**9**			**3**		**1**		**5**		**4**	
**Grade, n**												
**1**			**9**	**3**		**4**		**1**		**5**		**2**
**2**			**3**	**9**		**2**		**4**		**1**		**5**
**3**			**2**	**3**		**2**		**2**		**-**		**1**
**Inadequate for interpretation**			**3**	**3**		**3**		**3**		**-**		**-**
**Postoperative classification**												
**Higher stages, n**[*]	**35 (13.9%)**				**11 (10.4%)**		**10 (24.4%)**		**6 (8.1%)**		**8 (26.6%)**	

[*] Higher stages: higher stages than postoperative Groups 1–4. Higher stages included stage 1A and grade 3, stage IB, stage II, and stage IIIC.
MI, myometrial invasion.
