# Supplementary material for: Prediction of final pathology depending on preoperative myometrial invasion and grade assessment in low-risk endometrial cancer patients: A Korean Gynecologic Oncology Group ancillary study
Source: PLoS One. 2024 Jun 27;19(6):e0305360. doi: 10.1371/journal.pone.0305360 (PMC11210801; doi:10.1371/journal.pone.0305360)
Supplement: S2 Table — (PDF) [file pone.0305360.s004.pdf]

			Preoperative D&C	
Patient number	Age	Menopause	Method	Histologic type
1	54	Yes	Dilatation/currettage	Endometrioid
2	55	Yes	Dilatation/currettage	Endometrioid
3	45	No	Dilatation/currettage	Endometrioid
4	62	Yes	Dilatation/currettage	Endometrioid
5	55	Yes	Dilatation/currettage	Endometrioid
6	51	Yes	Dilatation/currettage	Endometrioid
7	47	No	Dilatation/currettage	Endometrioid
8	45	No	Dilatation/currettage	Endometrioid
9	44	No	Dilatation/currettage	Endometrioid
10	61	Yes	Dilatation/currettage	Endometrioid
11	42	No	Dilatation/currettage	Endometrioid
12	54	No	Dilatation/currettage	Endometrioid
13	61	Yes	Dilatation/currettage	Endometrioid
14	66	Yes	Dilatation/currettage	Endometrioid
15	56	Yes	Dilatation/currettage	Endometrioid
16	41	No	Dilatation/currettage	Endometrioid
17	59	Yes	Dilatation/currettage	Endometrioid
18	52	Yes	Dilatation/currettage	Endometrioid
19	53	Yes	Hysteroscopy	Endometrioid
20	79	Yes	Dilatation/currettage	Endometrioid
21	49	Yes	Dilatation/currettage	Endometrioid
22	51	Yes	Hysteroscopy	Endometrioid
23	59	Yes	Pipelle biopsy	Endometrioid
24	45	Yes	Dilatation/currettage	Endometrioid
25	55	Yes	Dilatation/currettage	Endometrioid
26	45	No	Dilatation/currettage	Endometrioid
27	53	Yes	Pipelle biopsy	Endometrioid
28	55	Yes	Dilatation/currettage	Endometrioid
29	55	Yes	Dilatation/currettage	Endometrioid
30	72	Yes	Dilatation/currettage	Endometrioid
31	45	No	Dilatation/currettage	Endometrioid
32	52	No	Dilatation/currettage	Endometrioid
33	54	Yes	Dilatation/currettage	Endometrioid
34	75	Yes	Dilatation/currettage	Endometrioid
35	40	No	Dilatation/currettage	Endometrioid
36	69	Yes	Dilatation/currettage	Endometrioid
37	51	No	Dilatation/currettage	Endometrioid
38	37	No	Dilatation/currettage	Endometrioid
39	57	Yes	Dilatation/currettage	Endometrioid
40	42	No	Dilatation/currettage	Endometrioid
41	59	Yes	Dilatation/currettage	Endometrioid
42	61	Yes	Dilatation/currettage	Endometrioid
43	61	Yes	Dilatation/currettage	Endometrioid
44	41	No	Dilatation/currettage	Endometrioid
45	50	No	Dilatation/currettage	Endometrioid
46	47	No	Dilatation/currettage	Endometrioid
47	55	Yes	Dilatation/currettage	Endometrioid
48	52	Yes	Dilatation/currettage	Endometrioid
49	51	Yes	Dilatation/currettage	Endometrioid
50	40	No	Dilatation/currettage	Endometrioid
51	58	Yes	Dilatation/currettage	Endometrioid
52	53	Yes	Dilatation/currettage	Endometrioid
53	71	Yes	Dilatation/currettage	Endometrioid

54	52	Yes	Dilatation/currettage	Endometrioid
55	61	Yes	Dilatation/currettage	Endometrioid
56	53	Yes	Dilatation/currettage	Endometrioid
57	52	Yes	Dilatation/currettage	Endometrioid
58	56	Yes	Dilatation/currettage	Endometrioid
59	54	Yes	Dilatation/currettage	Endometrioid
60	52	Yes	Dilatation/currettage	Endometrioid
61	52	Yes	Dilatation/currettage	Endometrioid
62	44	No	Dilatation/currettage	Endometrioid
63	44	No	Dilatation/currettage	Endometrioid
64	64	Yes	Dilatation/currettage	Endometrioid
65	59	Yes	Dilatation/currettage	Endometrioid
66	37	No	Dilatation/currettage	Endometrioid
67	54	Yes	Dilatation/currettage	Endometrioid
68	37	No	Dilatation/currettage	Endometrioid
69	54	Yes	Dilatation/currettage	Endometrioid
70	53	Yes	Dilatation/currettage	Endometrioid
71	29	No	Dilatation/currettage	Endometrioid
72	66	No	Dilatation/currettage	Endometrioid
73	42	No	Dilatation/currettage	Endometrioid
74	57	Yes	Dilatation/currettage	Endometrioid
75	49	Yes	Dilatation/currettage	Endometrioid
76	67	No	Dilatation/currettage	Endometrioid
77	72	Yes	Dilatation/currettage	Endometrioid
78	49	Yes	Dilatation/currettage	Endometrioid
79	62	Yes	Dilatation/currettage	Endometrioid
80	56	Yes	Hysteroscopy	Endometrioid
81	53	No	Dilatation/currettage	Endometrioid
82	68	Yes	Dilatation/currettage	Endometrioid
83	48	Yes	Dilatation/currettage	Endometrioid
84	48	Yes	Dilatation/currettage	Endometrioid
85	35	No	Dilatation/currettage	Endometrioid
86	34	No	Dilatation/currettage	Endometrioid
87	50	No	Dilatation/currettage	Endometrioid
88	43	No	Dilatation/currettage	Endometrioid
89	53	Yes	Dilatation/currettage	Endometrioid
90	51	No	Dilatation/currettage	Endometrioid
91	44	Yes	Dilatation/currettage	Endometrioid
92	57	Yes	Dilatation/currettage	Endometrioid
93	54	Yes	Pipelle biopsy	Endometrioid
94	56	Yes	Dilatation/currettage	Endometrioid
95	40	No	Dilatation/currettage	Endometrioid
96	71	Yes	Dilatation/currettage	Endometrioid
97	38	No	Pipelle biopsy	Endometrioid
98	53	No	Hysteroscopy	Endometrioid
99	56	Yes	Dilatation/currettage	Endometrioid
100	41	No	Dilatation/currettage	Endometrioid
101	39	No	Dilatation/currettage	Endometrioid
102	62	Yes	Dilatation/currettage	Endometrioid
103	66	Yes	Dilatation/currettage	Endometrioid
104	45	No	Pipelle biopsy	Endometrioid
105	49	Yes	Dilatation/currettage	Endometrioid
106	70	Yes	Dilatation/currettage	Endometrioid
107	49	No	Dilatation/currettage	Endometrioid
108	48	No	Dilatation/currettage	Endometrioid
109	51	No	Dilatation/currettage	Endometrioid
110	55	Yes	Pipelle biopsy	Endometrioid
111	48	No	Dilatation/currettage	Endometrioid
112	53	Yes	Dilatation/currettage	Endometrioid

113	52	Yes	Pipelle biopsy	Endometrioid
114	52	Yes	Dilatation/currettage	Endometrioid
115	57	Yes	Pipelle biopsy	Endometrioid
116	61	Yes	Dilatation/currettage	Endometrioid
117	53	Yes	Dilatation/currettage	Endometrioid
118	71	Yes	Dilatation/currettage	Endometrioid
119	51	No	Dilatation/currettage	Endometrioid
120	56	Yes	Pipelle biopsy	Endometrioid
121	57	Yes	Dilatation/currettage	Endometrioid
122	54	Yes	Pipelle biopsy	Endometrioid
123	60	Yes	Dilatation/currettage	Endometrioid
124	43	No	Dilatation/currettage	Endometrioid
125	53	Yes	Dilatation/currettage	Endometrioid
126	63	Yes	Dilatation/currettage	Endometrioid
127	56	Yes	Dilatation/currettage	Endometrioid
128	55	Yes	Dilatation/currettage	Endometrioid
129	65	Yes	Dilatation/currettage	Endometrioid
130	58	Yes	Dilatation/currettage	Endometrioid
131	36	No	Dilatation/currettage	Endometrioid
132	52	No	Dilatation/currettage	Endometrioid
133	55	No	Dilatation/currettage	Endometrioid
134	46	No	Dilatation/currettage	Endometrioid
135	56	Yes	Dilatation/currettage	Endometrioid
136	56	Yes	Dilatation/currettage	Endometrioid
137	56	Yes	Dilatation/currettage	Endometrioid
138	51	Yes	Dilatation/currettage	Endometrioid
139	51	Yes	Dilatation/currettage	Endometrioid
140	51	Yes	Dilatation/currettage	Endometrioid
141	48	Yes	Dilatation/currettage	Endometrioid
142	48	Yes	Dilatation/currettage	Endometrioid
143	52	Yes	Dilatation/currettage	Endometrioid
144	52	No	Dilatation/currettage	Endometrioid
145	45	No	Dilatation/currettage	Endometrioid
146	61	Yes	Dilatation/currettage	Endometrioid
147	43	No	Dilatation/currettage	Endometrioid
148	30	No	Dilatation/currettage	Endometrioid
149	50	No	Dilatation/currettage	Endometrioid
150	57	Yes	Dilatation/currettage	Endometrioid
151	47	No	Dilatation/currettage	Endometrioid
152	43	No	Hysteroscopy	Endometrioid
153	40	No	Dilatation/currettage	Endometrioid
154	41	No	Dilatation/currettage	Endometrioid
155	52	No	Dilatation/currettage	Endometrioid
156	51	Yes	Dilatation/currettage	Endometrioid
157	37	No	Dilatation/currettage	Endometrioid
158	58	Yes	Dilatation/currettage	Endometrioid
159	67	Yes	Dilatation/currettage	Endometrioid
160	48	No	Dilatation/currettage	Endometrioid
161	66	Yes	Dilatation/currettage	Endometrioid
162	49	No	Dilatation/currettage	Endometrioid
163	46	No	Dilatation/currettage	Endometrioid
164	49	Yes	Dilatation/currettage	Endometrioid
165	47	No	Dilatation/currettage	Endometrioid
166	60	Yes	Dilatation/currettage	Endometrioid
167	52	Yes	Hysteroscopy	Endometrioid
168	46	Yes	Dilatation/currettage	Endometrioid
169	36	No	Dilatation/currettage	Endometrioid
170	62	Yes	Pipelle biopsy	Endometrioid
171	58	Yes	Dilatation/currettage	Endometrioid

172	50	Yes	Dilatation/currettage	Endometrioid
173	46	No	Dilatation/currettage	Endometrioid
174	32	No	Dilatation/currettage	Endometrioid
175	50	No	Dilatation/currettage	Endometrioid
176	63	Yes	Dilatation/currettage	Endometrioid
177	56	Yes	Dilatation/currettage	Endometrioid
178	60	Yes	Dilatation/currettage	Endometrioid
179	50	No	Dilatation/currettage	Endometrioid
180	51	Yes	Dilatation/currettage	Endometrioid
181	75	Yes	Dilatation/currettage	Endometrioid
182	53	Yes	Dilatation/currettage	Endometrioid
183	62	Yes	Dilatation/currettage	Endometrioid
184	56	Yes	Dilatation/currettage	Endometrioid
185	52	Yes	Dilatation/currettage	Endometrioid
186	58	No	Dilatation/currettage	Endometrioid
187	48	No	Dilatation/currettage	Endometrioid
188	55	Yes	Dilatation/currettage	Endometrioid
189	36	No	Dilatation/currettage	Endometrioid
190	56	Yes	Dilatation/currettage	Endometrioid
191	48	No	Dilatation/currettage	Endometrioid
192	49	Yes	Dilatation/currettage	Endometrioid
193	53	Yes	Dilatation/currettage	Endometrioid
194	51	No	Dilatation/currettage	Endometrioid
195	43	No	Dilatation/currettage	Endometrioid
196	40	No	Dilatation/currettage	Endometrioid
197	37	No	Dilatation/currettage	Endometrioid
198	53	Yes	Dilatation/currettage	Endometrioid
199	53	No	Dilatation/currettage	Endometrioid
200	72	Yes	Dilatation/currettage	Endometrioid
201	39	No	Dilatation/currettage	Endometrioid
202	32	No	Dilatation/currettage	Endometrioid
203	45	No	Dilatation/currettage	Endometrioid
204	51	No	Dilatation/currettage	Endometrioid
205	46	No	Dilatation/currettage	Endometrioid
206	51	Yes	Dilatation/currettage	Endometrioid
207	63	Yes	Dilatation/currettage	Endometrioid
208	46	Yes	Dilatation/currettage	Endometrioid
209	54	Yes	Dilatation/currettage	Endometrioid
210	60	Yes	Dilatation/currettage	Endometrioid
211	53	No	Dilatation/currettage	Endometrioid
212	57	Yes	Dilatation/currettage	Endometrioid
213	47	No	Dilatation/currettage	Endometrioid
214	44	No	Dilatation/currettage	Endometrioid
215	75	Yes	Dilatation/currettage	Endometrioid
216	49	No	Dilatation/currettage	Endometrioid
217	59	Yes	Dilatation/currettage	Endometrioid
218	61	Yes	Dilatation/currettage	Endometrioid
219	66	Yes	Dilatation/currettage	Endometrioid
220	55	Yes	Dilatation/currettage	Endometrioid
221	43	No	Dilatation/currettage	Endometrioid
222	37	No	Dilatation/currettage	Endometrioid
223	55	Yes	Dilatation/currettage	Endometrioid
224	58	Yes	Dilatation/currettage	Endometrioid
225	50	No	Dilatation/currettage	Endometrioid
226	51	Yes	Dilatation/currettage	Endometrioid
227	35	No	Dilatation/currettage	Endometrioid
228	51	Yes	Dilatation/currettage	Endometrioid
229	54	Yes	Dilatation/currettage	Endometrioid
230	51	Yes	Dilatation/currettage	Endometrioid

231	56	Yes	Dilatation/currettage	Endometrioid
232	51	No	Dilatation/currettage	Endometrioid
233	54	No	Dilatation/currettage	Endometrioid
234	53	Yes	Dilatation/currettage	Endometrioid
235	54	Yes	Dilatation/currettage	Endometrioid
236	45	No	Dilatation/currettage	Endometrioid
237	52	Yes	Dilatation/currettage	Endometrioid
238	54	Yes	Dilatation/currettage	Endometrioid
239	49	Yes	Dilatation/currettage	Endometrioid
240	66	Yes	Dilatation/currettage	Endometrioid
241	52	No	Dilatation/currettage	Endometrioid
242	68	Yes	Dilatation/currettage	Endometrioid
243	57	Yes	Dilatation/currettage	Endometrioid
244	53	Yes	Pipelle biopsy	Endometrioid
245	51	Yes	Dilatation/currettage	Endometrioid
246	51	No	Dilatation/currettage	Endometrioid
247	46	No	Pipelle biopsy	Endometrioid
248	52	No	Pipelle biopsy	Endometrioid
249	50	No	Pipelle biopsy	Endometrioid
250	62	Yes	Pipelle biopsy	Endometrioid
251	51	No	Pipelle biopsy	Endometrioid

	Preoperative	Preoperative MRI		
Grade	CA125 (IU/ml)	Myometrial invasion depth	Tumor size (largest diameter)	Route
I	14.94	Less than 50% (< 1/2)	3	Laparoscopic
I	20.38	None	1.7	Laparoscopic
I	43.47	Less than 50% (< 1/2)	1.25	Laparoscopic
I	13.83	Less than 50% (< 1/2)	4.7	Laparoscopic
I	9.02	Less than 50% (< 1/2)	1.3	Laparoscopic
I	15.84	Less than 50% (< 1/2)	2.3	Laparoscopic
I	34.8	Less than 50% (< 1/2)	5.5	Laparoscopic
I	11.02	Less than 50% (< 1/2)	0.6	Laparoscopic
I	28.95	Less than 50% (< 1/2)	2.3	Laparoscopic
I	14.8	Less than 50% (< 1/2)	2	Laparoscopic
I	10.16	Less than 50% (< 1/2)	1.3	Laparoscopic
I	23.08	Less than 50% (< 1/2)	.	Laparoscopic
II	13.31	Less than 50% (< 1/2)	3.7	Laparoscopic
I	18.74	Less than 50% (< 1/2)	1.7	Laparoscopic
I	16.5	None	4.2	Laparoscopic
I	23	None	1.5	Laparoscopic
I	8.5	None	2.9	Open (abdominal)
I	44.3	None	2.2	Robotic
I	13.5	None	.	Laparoscopic
I	7.8	Less than 50% (< 1/2)	3.3	Open (abdominal)
II	26.5	None	.	Robotic
I	11.5	None	.	Robotic
II	14.6	Less than 50% (< 1/2)	.	Laparoscopic
II	21.1	None	5	Open (abdominal)
I	13.2	None	2.8	Laparoscopic
II	19.7	None	0	Laparoscopic
I	17.8	None	2.5	Laparoscopic
II	7.69	None	0	Laparoscopic
II	19	None	0	Laparoscopic
I	40.05	Less than 50% (< 1/2)	1.8	Laparoscopic
I	40.57	Less than 50% (< 1/2)	.	Open (abdominal)
I	20.15	None	2.9	Laparoscopic
I	18.46	None	5.8	Open (abdominal)
I	8.11	None	.	Laparoscopic
I	37.12	None	.	Laparoscopic
I	39.56	None	.	Laparoscopic
I	20.01	None	.	Laparoscopic
I	18.78	None	0	Laparoscopic
I	42.31	Less than 50% (< 1/2)	2.5	Laparoscopic
I	16.28	None	.	Open (abdominal)
I	33.98	None	.	Open (abdominal)
II	16.31	None	.	Open (abdominal)
I	40.61	None	.	Open (abdominal)
I	30.79	Less than 50% (< 1/2)	.	Laparoscopic
II	48.6	None	.	Laparoscopic
I	29.4	Less than 50% (< 1/2)	2	Laparoscopic
I	6.5	Less than 50% (< 1/2)	2	Laparoscopic
II	17.9	Less than 50% (< 1/2)	2.5	Laparoscopic
II	29.8	None	.	Laparoscopic
I	20.2	None	.	Laparoscopic
I	20.6	None	.	Laparoscopic
I	15.3	None	.	Laparoscopic
II	16.8	Less than 50% (< 1/2)	2	Laparoscopic

I	25.1	None	.	Laparoscopic
II	14.6	Less than 50% (< 1/2)	1.5	Laparoscopic
II	21.3	Less than 50% (< 1/2)	.	Laparoscopic
II	20	None	0.1	Laparoscopic
II	19.7	Less than 50% (< 1/2)	4	Laparoscopic
II	31.1	None	.	Laparoscopic
II	21.7	None	.	Laparoscopic
I	13.3	Less than 50% (< 1/2)	.	Laparoscopic
II	24.9	Less than 50% (< 1/2)	.	Laparoscopic
I	19.9	Less than 50% (< 1/2)	.	Laparoscopic
I	33.8	Less than 50% (< 1/2)	4	Laparoscopic
I	7	Less than 50% (< 1/2)	.	Laparoscopic
II	19.8	None	2	Laparoscopic
I	24.7	None	.	Laparoscopic
II	17.9	Less than 50% (< 1/2)	2	Open (abdominal)
I	19.8	Less than 50% (< 1/2)	.	Laparoscopic
II	19.6	Less than 50% (< 1/2)	3	Laparoscopic
II	9.1	Less than 50% (< 1/2)	4	Laparoscopic
I	19.68	Less than 50% (< 1/2)	1	Open (abdominal)
I	13.93	Less than 50% (< 1/2)	2.8	Laparoscopic
I	13.64	Less than 50% (< 1/2)	.	Open (abdominal)
I	16.75	Less than 50% (< 1/2)	5	Open (abdominal)
I	13.1	None	2.6	Laparoscopic
I	31	None	2.9	Laparoscopic
II	23	None	.	Laparoscopic
I	7	None	.	Laparoscopic
I	15.6	None	1	Laparoscopic
II	11	None	3	Laparoscopic
II	25	None	.	Laparoscopic
II	81	Less than 50% (< 1/2)	7.58	Laparoscopic
II	16	None	1.3	Laparoscopic
II	13	None	3.4	Laparoscopic
II	17	None	1	Laparoscopic
I	19	None	1	Laparoscopic
I	21	None	2.1	Laparoscopic
I	12	Less than 50% (< 1/2)	3.5	Laparoscopic
II	19	None	0	Laparoscopic
I	18	None	.	Laparoscopic
II	10.3	Less than 50% (< 1/2)	1.7	Open (abdominal)
I	6.1	None	1.7	Open (abdominal)
I	5.2	None	1	Laparoscopic
II	4.5	Less than 50% (< 1/2)	.	Laparoscopic
I	23.8	None	.	Robotic
I	7.3	None	1.2	Laparoscopic
I	5.7	None	1	Laparoscopic
I	3.9	None	1.1	Laparoscopic
I	5.1	Less than 50% (< 1/2)	4.5	Laparoscopic
I	3.9	None	3.6	Robotic
I	1.1	None	0.9	Open (abdominal)
I	8.9	None	.	Laparoscopic
I	3.5	None	.	Open (abdominal)
I	9.2	Less than 50% (< 1/2)	5	Laparoscopic
I	5.4	None	3	Laparoscopic
I	22.5	None	3	Laparoscopic
I	12.1	None	5.7	Laparoscopic
I	3.6	None	1.5	Laparoscopic
I	8.4	None	2	Laparoscopic
I	4.2	Less than 50% (< 1/2)	2.6	Robotic
I	6.7	None	3.1	Laparoscopic

I	120.1	None	2.7	Laparoscopic
I	9.7	None	.	Laparoscopic
II	7.6	None	2.9	Laparoscopic
I	3.8	Less than 50% (< 1/2)	2	Robotic
I	5.4	None	2.2	Laparoscopic
II	7.6	None	.	Laparoscopic
I	18.2	Less than 50% (< 1/2)	.	Robotic
II	7.6	None	0.8	Laparoscopic
I	3	Less than 50% (< 1/2)	2.4	Open (abdominal)
I	17.9	None	.	Laparoscopic
II	11.8	None	1.2	Laparoscopic
I	7.5	Less than 50% (< 1/2)	3.5	Open (abdominal)
I	6.7	None	4.2	Laparoscopic
I	7.1	Less than 50% (< 1/2)	2.8	Laparoscopic
II	1.9	None	4.1	Laparoscopic
I	6.5	Less than 50% (< 1/2)	0	Laparoscopic
I	13.6	None	1.6	Laparoscopic
II	14.1	None	.	Laparoscopic
I	12.1	None	.	Laparoscopic
I	5.3	None	.	Laparoscopic
I	16.6	None	.	Laparoscopic
II	19.9	Less than 50% (< 1/2)	1.5	Open (abdominal)
II	23.4	None	0	Laparoscopic
I	15.3	None	0	Laparoscopic
I	19.1	None	0	Laparoscopic
I	9.2	Less than 50% (< 1/2)	0	Laparoscopic
I	22.8	Less than 50% (< 1/2)	0	Laparoscopic
II	42.4	Less than 50% (< 1/2)	0	Laparoscopic
I	44.6	None	0	Laparoscopic
I	44.6	None	0	Laparoscopic
I	11.5	None	0	Laparoscopic
I	10.7	None	0	Laparoscopic
II	18.7	Less than 50% (< 1/2)	1.2	Laparoscopic
I	5	Less than 50% (< 1/2)	.	Laparoscopic
I	21.9	None	0	Laparoscopic
I	7.7	None	0	Laparoscopic
I	8.3	None	0	Laparoscopic
I	5.1	Less than 50% (< 1/2)	2.4	Laparoscopic
I	14	None	0	Laparoscopic
I	13	Less than 50% (< 1/2)	1.8	Laparoscopic
I	14.2	None	.	Laparoscopic
I	34	None	.	Laparoscopic
I	11.7	Less than 50% (< 1/2)	1.3	Open (abdominal)
I	5.5	None	.	Laparoscopic
II	18.5	None	.	Laparoscopic
II	16.2	None	.	Laparoscopic
I	12.2	Less than 50% (< 1/2)	1	Laparoscopic
I	26	Less than 50% (< 1/2)	3.3	Robotic
I	7.9	Less than 50% (< 1/2)	3	Laparoscopic
I	18.2	None	.	Open (abdominal)
I	17.6	None	.	Laparoscopic
I	15.1	Less than 50% (< 1/2)	.	Robotic
I	17.2	None	.	Robotic
II	10.9	Less than 50% (< 1/2)	1.9	Open (abdominal)
II	11.1	Less than 50% (< 1/2)	2	Laparoscopic
I	53.1	Less than 50% (< 1/2)	4	Laparoscopic
I	9.8	None	.	Laparoscopic
II	9.8	Less than 50% (< 1/2)	.	Laparoscopic
II	18.7	Less than 50% (< 1/2)	2.9	Laparoscopic

I	5.9	Less than 50% (< 1/2)	3	Laparoscopic
I	12.8	Less than 50% (< 1/2)	.	Laparoscopic
I	11.6	None	.	Laparoscopic
I	7.8	None	0	Laparoscopic
I	20.1	None	.	Laparoscopic
I	22.7	None	4.1	Laparoscopic
I	13.5	Less than 50% (< 1/2)	2.3	Laparoscopic
I	21.2	None	.	Laparoscopic
I	16.6	Less than 50% (< 1/2)	.	Laparoscopic
I	6.6	Less than 50% (< 1/2)	4	Laparoscopic
II	35.2	Less than 50% (< 1/2)	.	Laparoscopic
I	18.7	Less than 50% (< 1/2)	3.5	Open (abdominal)
I	14.3	Less than 50% (< 1/2)	.	Laparoscopic
II	9.4	None	1.3	Laparoscopic
II	6.1	None	4	Laparoscopic
I	5.3	Less than 50% (< 1/2)	1	Laparoscopic
I	495	Less than 50% (< 1/2)	8.3	Open (abdominal)
I	4.5	None	5	Laparoscopic
II	10.8	Less than 50% (< 1/2)	.	Laparoscopic
II	6.9	Less than 50% (< 1/2)	3.2	Laparoscopic
I	5.2	None	.	Laparoscopic
II	12.7	None	.	Laparoscopic
I	34.5	Less than 50% (< 1/2)	.	Laparoscopic
I	6.7	None	1.8	Laparoscopic
I	11.5	None	.	Laparoscopic
I	9.1	Less than 50% (< 1/2)	.	Laparoscopic
II	213	Less than 50% (< 1/2)	3	Laparoscopic
I	8.5	None	.	Laparoscopic
I	5.5	Less than 50% (< 1/2)	1	Laparoscopic
I	5	None	1.3	Laparoscopic
I	30.7	Less than 50% (< 1/2)	4.4	Laparoscopic
II	35.9	None	2	Laparoscopic
I	10.2	None	.	Laparoscopic
I	8.7	None	.	Open (abdominal)
II	7.7	None	.	Open (abdominal)
II	20.6	None	.	Open (abdominal)
I	9	Less than 50% (< 1/2)	.	Open (abdominal)
II	10.9	Less than 50% (< 1/2)	.	Laparoscopic
I	5.4	None	.	Laparoscopic
I	8.6	Less than 50% (< 1/2)	3.2	Robotic
I	42.31	Less than 50% (< 1/2)	2.5	Laparoscopic
I	13.81	None	0	Laparoscopic
I	20.8	Less than 50% (< 1/2)	.	Laparoscopic
II	24.63	None	.	Laparoscopic
I	16.22	None	0	Laparoscopic
I	8.79	None	2	Laparoscopic
I	15.16	None	0.6	Laparoscopic
I	132.8	None	3	Laparoscopic
I	21.61	None	1.5	Laparoscopic
I	49.43	None	.	Laparoscopic
I	18.04	None	2.6	Laparoscopic
II	12.11	None	0	Open (abdominal)
I	32.81	Less than 50% (< 1/2)	.	Laparoscopic
I	26.63	None	0	Laparoscopic
II	9	None	0	Laparoscopic
I	12.21	None	0	Laparoscopic
I	10.79	None	2	Laparoscopic
I	9.47	Less than 50% (< 1/2)	3	Open (abdominal)
II	11.41	None	0	Laparoscopic

II	30.94	None	.	Open (abdominal)
II	31.22	None	.	Laparoscopic
II	11.08	Less than 50% (< 1/2)	2.6	Laparoscopic
I	7.22	None	0	Laparoscopic
I	6.8	None	5.5	Open (abdominal)
I	11.9	None	1.8	Laparoscopic
I	15.8	None	1	Laparoscopic
I	12.3	None	1.45	Laparoscopic
I	18.3	None	0.6	Open (abdominal)
I	18.9	None	0	Laparoscopic
I	36.7	Less than 50% (< 1/2)	17	Laparoscopic
I	16.7	Less than 50% (< 1/2)	3.9	Open (abdominal)
II	11.86	Less than 50% (< 1/2)	1.9	Laparoscopic
I	29.97	Less than 50% (< 1/2)	1.5	Laparoscopic
I	8.93	None	.	Laparoscopic
I	9.76	Less than 50% (< 1/2)	0.5	Laparoscopic
I	16.4	Less than 50% (< 1/2)	3	Laparoscopic
I	14.6	Less than 50% (< 1/2)	2.9	Laparoscopic
II	28.4	None	.	Laparoscopic
II	14.1	Less than 50% (< 1/2)	1	Laparoscopic
II	39.21	Less than 50% (< 1/2)	1.5	Laparoscopic

Postoperative patholog

Stage	Histologic diagnosis	Grade
Ia	Endometrioid	I
Ia	Endometrioid	I
Ia	Endometrioid	I
Ia	Endometrioid	I
Ia	Endometrioid	I
Ia	Endometrioid	I
Ia	Endometrioid	I
Ia	Endometrioid	I
Ia	Endometrioid	I
Ia	Endometrioid	I
Ia	Endometrioid	II
Ia	Endometrioid	I
Ia	Endometrioid	II
Ia	Endometrioid	I
Ia	Endometrioid	I
Ia	Endometrioid	I
Ia	Endometrioid	III
Ia	Endometrioid	I
Ia	Endometrioid	I
Ia	Endometrioid	I
Ia	Endometrioid	II
Ia	Endometrioid	I
II	Endometrioid	II
Ia	Endometrioid	I
Ia	Endometrioid	I
Ia	Endometrioid	II
Ia	Endometrioid	I
Ia	Squamous or adenosquamous	II
IIIc	Endometrioid	II
Ia	Endometrioid	I
Ia	Endometrioid	I
Ia	Endometrioid	I
Ib	Endometrioid	II
Ia	Endometrioid	I
Ia	Endometrioid	I
Ia	Endometrioid	I
Ia	Endometrioid	I
Ia	Endometrioid	I
Ia	Endometrioid	I
Ia	Endometrioid	II
Ia	Endometrioid	I
Ia	Endometrioid	I
Ia	Endometrioid	I
Ia	Endometrioid	I
Ia	Endometrioid	I
Ia	Endometrioid	I
Ia	Endometrioid	I
Ia	Endometrioid	I
Ia	Endometrioid	I
Ia	Endometrioid	I
Ia	Endometrioid	II
Ia	Endometrioid	I
Ia	Endometrioid	II

Ia	Endometrioid	I
Ia	Endometrioid	I
Ib	Endometrioid	I
IIIc	Endometrioid	I
Ib	Endometrioid	II
Ia	Endometrioid	I
Ia	Endometrioid	Inadequate for interpretation
Ia	Endometrioid	I
Ia	Endometrioid	I
Ia	Endometrioid	I
Ia	Endometrioid	I
Ib	Endometrioid	I
Ia	Endometrioid	I
Ia	Endometrioid	I
Ia	Endometrioid	II
Ia	Endometrioid	I
Ib	Endometrioid	I
Ia	Endometrioid	I
Ia	Endometrioid	I
Ia	Endometrioid	I
Ia	Endometrioid	II
Ib	Endometrioid	II
Ia	Endometrioid	I
Ib	Endometrioid	II
Ia	Endometrioid	I
Ia	Endometrioid	Inadequate for interpretation
Ia	Endometrioid	I
Ia	Endometrioid	I
Ia	Endometrioid	II
II	Endometrioid	II
Ia	Endometrioid	II
Ia	Endometrioid	II
II	Endometrioid	II
Ia	Endometrioid	I
Ia	Endometrioid	II
Ia	Endometrioid	I
Ia	Endometrioid	I
Ia	Endometrioid	I
Ia	Endometrioid	III
Ia	Endometrioid	I
Ia	Endometrioid	I
Ia	Endometrioid	I
IIIc	Mixed (with endometrioid)	I
Ia	Endometrioid	I
Ia	Endometrioid	I
Ia	Endometrioid	I
Ia	Endometrioid	I
Ia	Endometrioid	I
Ia	Endometrioid	I
Ia	Endometrioid	I
Ia	Endometrioid	I
Ia	Endometrioid	I
Ia	Endometrioid	I
Ia	Endometrioid	I
Ia	Endometrioid	I
Ia	Endometrioid	II
Ia	Endometrioid	I
Ia	Endometrioid	I
Ia	Endometrioid	I

Ia	Endometrioid	I
Ia	Endometrioid	I
Ia	Endometrioid	I
Ia	Endometrioid	I
Ia	Endometrioid	I
Ia	Endometrioid	II
Ia	Endometrioid	I
Ia	Endometrioid	I
Ia	Endometrioid	I
Ia	Endometrioid	I
Ia	Endometrioid	I
Ia	Endometrioid	II
Ia	Endometrioid	I
Ia	Endometrioid	I
Ia	Endometrioid	Inadequate for interpretation
Ia	Endometrioid	I
Ia	Endometrioid	I
Ia	Endometrioid	II
Ia	Endometrioid	Inadequate for interpretation
Ia	Endometrioid	II
Ia	Endometrioid	I
Ia	Endometrioid	II
Ia	Endometrioid	I
Ia	Endometrioid	II
Ia	Endometrioid	I
Ia	Endometrioid	I
Ia	Endometrioid	I
Ia	Endometrioid	I
Ia	Endometrioid	I
Ia	Endometrioid	I
Ia	Endometrioid	I
Ia	Endometrioid	II
Ia	Endometrioid	II
Ia	Endometrioid	I
Ia	Endometrioid	I
Ia	Endometrioid	I
Ia	Endometrioid	III
Ib	Endometrioid	I
Ia	Endometrioid	I
Ia	Endometrioid	II
Ia	Endometrioid	I
Ia	Endometrioid	I
Ia	Endometrioid	I
IIIc	Endometrioid	I
Ia	Endometrioid	II
Ia	Endometrioid	I
Ia	Endometrioid	I
Ia	Endometrioid	I
Ib	Endometrioid	I
Ia	Endometrioid	I
Ia	Endometrioid	I
II	Endometrioid	I
Ia	Endometrioid	I
Ia	Endometrioid	II
Ia	Endometrioid	I
Ia	Endometrioid	I
Ia	Endometrioid	I
Ia	Endometrioid	II
Ia	Endometrioid	II

Ia	Endometrioid	II
Ia	Endometrioid	I
Ia	Endometrioid	I
Ia	Endometrioid	I
Ia	Endometrioid	I
Ia	Endometrioid	II
Ib	Endometrioid	I
Ia	Endometrioid	I
Ia	Endometrioid	I
Ia	Endometrioid	I
Ib	Endometrioid	II
Ia	Endometrioid	I
Ia	Mixed (with endometrioid)	I
Ia	Endometrioid	III
Ia	Endometrioid	III
Ia	Endometrioid	I
Ia	Endometrioid	I
Ia	Endometrioid	I
Ia	Endometrioid	II
II	Endometrioid	II
Ia	Endometrioid	I
Ia	Endometrioid	II
Ia	Endometrioid	II
Ia	Endometrioid	I
Ia	Endometrioid	I
Ia	Endometrioid	I
Ia	Endometrioid	II
Ia	Endometrioid	I
Ia	Endometrioid	I
Ia	Endometrioid	I
Ia	Endometrioid	I
II	Endometrioid	II
Ia	Endometrioid	I
Ia	Endometrioid	I
Ia	Endometrioid	II
Ia	Endometrioid	II
Ia	Endometrioid	I
Ia	Endometrioid	II
Ia	Endometrioid	I
Ia	Endometrioid	I
Ia	Endometrioid	I
Ia	Endometrioid	I
Ia	Endometrioid	I
Ia	Endometrioid	II
Ia	Endometrioid	I
Ia	Endometrioid	II
Ia	Endometrioid	I
Ib	Endometrioid	I
Ia	Endometrioid	I
Ia	Endometrioid	I
Ia	Endometrioid	I
Ia	Endometrioid	Inadequate for interpretation
Ia	Endometrioid	II
Ia	Endometrioid	Inadequate for interpretation
Ia	Endometrioid	II
IIIc	Endometrioid	I
Ia	Endometrioid	II
Ia	Endometrioid	II
Ia	Endometrioid	I

Ia	Endometrioid	II
IIIc	Endometrioid	II
Ia	Endometrioid	II
Ia	Endometrioid	II
Ia	Endometrioid	I
Ia	Endometrioid	I
Ia	Endometrioid	I
Ia	Endometrioid	I
Ia	Endometrioid	I
Ia	Endometrioid	I
Ia	Endometrioid	II
Ia	Endometrioid	I
Ia	Endometrioid	II
Ia	Endometrioid	I
Ia	Endometrioid	I
Ia	Endometrioid	I
Ia	Endometrioid	I
Ia	Endometrioid	I
Ia	Endometrioid	II
Ia	Endometrioid	II
Ia	Endometrioid	II

ic findings

Myometrial invasion depth	Tumor size (largest diameter)	Extrauterine involvement	Lymphovascular space invasion (LVSI)	Metastasis of pelvic lymph node
Less than 50%	2.5	none	No	0
Less than 50%	1	none	No	0
Less than 50%	3.8	none	No	0
Less than 50%	4	none	No	0
Less than 50%	2	none	No	0
Less than 50%	2.5	none	No	0
Less than 50%	5	none	No	0
Less than 50%	0.8	none	No	0
Less than 50%	0.2	none	No	0
Less than 50%	2	none	No	0
Less than 50%	1.2	none	No	0
Less than 50%	1.5	none	No	0
Less than 50%	4.7	none	No	0
Less than 50%	3	none	No	0
None	1.3	none	No	0
None	0.3	none	No	0
Less than 50%	3.7	none	Yes	0
None	2.2	none	No	0
Less than 50%	0	none	No	0
Less than 50%	0	none	No	0
Less than 50%	0	none	No	0
Less than 50%	0	none	No	0
Less than 50%	0	Cervix	No	0
Less than 50%	5.5	none	No	0
None	3	none	No	0
None	0	none	No	0
Less than 50%	1.5	none	No	0
None	0	none	No	0
None	0	none	No	yes
Less than 50%	2.2	none	No	0
Less than 50%	4.5	none	No	0
Less than 50%	3.5	none	No	0
More than 50%	4.6	none	No	0
Less than 50%	3.5	none	No	0
Less than 50%	4	none	No	0
Less than 50%	2	none	No	0
None	4	none	No	0
Less than 50%	4	none	No	0
Less than 50%	2.8	none	Yes	0
Less than 50%	0.5	none	No	0
Less than 50%	3.2	none	No	0
Less than 50%	1.4	none	No	0
None	0	none	No	0
Less than 50%	4.5	none	Yes	0
Less than 50%	3	none	Yes	0
Less than 50%	4.2	none	No	0
Less than 50%	2.6	none	No	0
Less than 50%	2.3	none	No	0
None	2.2	none	No	0
None	0.5	none	Yes	0
Less than 50%	1.8	none	No	0
None	1.1	none	No	0
Less than 50%	3	none	Yes	0

Less than 50%	1.5	none	No	0
Less than 50%	1.6	none	Yes	0
More than 50%	3.1	none	Yes	0
Less than 50%	0.9	none	No	yes
More than 50%	4	none	No	0
Less than 50%	2.5	none	No	0
None	0	none	No	0
Less than 50%	4.5	none	Yes	0
None	0.8	none	No	0
None	1	none	No	0
Less than 50%	4	none	No	0
More than 50%	4	none	No	0
Less than 50%	0.3	none	No	0
Less than 50%	0.4	none	No	0
None	0	none	No	0
Less than 50%	2.4	none	No	0
More than 50%	2.2	none	Yes	0
Less than 50%	2.8	none	No	0
Less than 50%	5.8	none	No	0
Less than 50%	2.8	none	No	0
Less than 50%	1.7	none	No	0
More than 50%	4.2	none	No	0
Less than 50%	3.1	none	No	0
More than 50%	4.3	none	No	0
Less than 50%	0.9	none	No	0
None	0.3	none	No	0
None	1.1	none	No	0
Less than 50%	3.5	none	No	0
Less than 50%	4.5	none	Yes	0
Less than 50%	5	Cervix	No	0
Less than 50%	2.2	none	No	0
Less than 50%	2.2	none	No	0
Less than 50%	2.2	Cervix	Yes	0
None	0.8	none	No	0
None	2.3	none	No	0
Less than 50%	3.5	none	No	0
None	1.9	none	No	0
None	0.7	none	No	0
None	1.5	none	No	0
Less than 50%	1.5	none	No	0
Less than 50%	0.6	none	No	0
None	0.8	none	No	0
Less than 50%	2.5	none	Yes	yes
None	1.8	none	No	0
None	0	none	No	0
Less than 50%	1	none	No	0
None	4.1	none	No	0
None	3.3	none	No	0
Less than 50%	1.5	none	No	0
None	3.2	none	No	0
Less than 50%	0	none	No	0
None	3	none	No	0
Less than 50%	0.6	none	No	0
Less than 50%	1.5	none	No	0
None	2	none	No	0
Less than 50%	1.8	none	No	0
None	2.3	none	No	0
Less than 50%	2.5	none	No	0
Less than 50%	2.1	none	No	0

None	2.8	none	No	0
Less than 50%	1.5	none	No	0
None	2.5	none	No	0
Less than 50%	2.2	none	No	0
Less than 50%	3.4	none	No	0
Less than 50%	1.5	none	yes	0
Less than 50%	2.5	none	No	0
Less than 50%	1	none	No	0
Less than 50%	3	none	No	0
Less than 50%	1.8	none	No	0
Less than 50%	2.7	none	No	0
Less than 50%	3	none	No	0
None	4.5	none	No	0
Less than 50%	3	none	No	0
Less than 50%	2	none	No	0
Less than 50%	1.8	none	No	0
Less than 50%	0	none	No	0
Less than 50%	2.2	none	No	0
None	0	none	No	0
None	0	none	No	0
None	5.5	none	No	0
Less than 50%	5.5	none	No	0
None	5.5	none	No	0
None	0	none	No	0
Less than 50%	3.5	none	No	0
None	3	none	No	0
None	0	none	No	0
None	0.8	none	No	0
None	0	none	No	0
None	0	none	No	0
None	0.8	none	No	0
None	1.8	none	No	0
Less than 50%	2.3	none	No	0
Less than 50%	2.3	none	No	0
None	3.1	none	No	0
None	2.7	none	No	0
None	1.2	none	No	0
More than 50%	3.8	none	No	0
None	5	none	No	0
Less than 50%	2.5	none	No	0
None	6	none	No	0
None	0	none	No	0
Less than 50%	2.5	none	No	0
Less than 50%	2.5	none	No	yes
None	0	none	No	0
Less than 50%	1.5	none	No	0
Less than 50%	1.2	none	No	0
None	3	none	No	0
More than 50%	3.5	none	No	0
None	0	none	No	0
None	0	none	No	0
Less than 50%	2.4	Cervix	No	0
None	0	none	No	0
Less than 50%	2.5	none	No	0
Less than 50%	0	none	No	0
None	4	none	No	0
None	0.7	none	No	0
Less than 50%	3.5	none	No	0
Less than 50%	3.7	none	No	0

Less than 50%	4	none	No	0
None	1.5	none	No	0
None	0	none	No	0
None	0.6	none	No	0
Less than 50%	3	none	No	0
Less than 50%	4.8	none	No	0
More than 50%	2.1	none	No	0
Less than 50%	3.1	none	No	0
None	0	none	No	0
Less than 50%	1.9	none	No	0
More than 50%	0	none	Yes	0
Less than 50%	3.5	none	No	0
Less than 50%	0	none	No	0
Less than 50%	1.5	none	Yes	0
Less than 50%	4.3	none	No	0
None	2	none	No	0
Less than 50%	8.2	none	No	0
Less than 50%	5	none	No	0
Less than 50%	1.8	none	No	0
Less than 50%	3.2	Cervix	No	0
None	0	none	No	0
Less than 50%	2.8	none	No	0
Less than 50%	4.2	none	No	0
None	5	none	No	0
Less than 50%	0.7	none	No	0
Less than 50%	1	none	No	0
Less than 50%	1.7	none	Yes	0
None	1	none	No	0
Less than 50%	1.5	none	No	0
Less than 50%	2.2	none	No	0
None	0	none	No	0
None	2	Cervix	No	0
Less than 50%	3	none	No	0
None	1	none	No	0
Less than 50%	1	none	No	0
None	0	none	No	0
None	2.5	none	No	0
Less than 50%	0.6	none	No	0
None	0	none	No	0
None	2.2	none	No	0
Less than 50%	2.8	none	Yes	0
Less than 50%	2.1	none	No	0
Less than 50%	1.5	none	No	0
None	0	none	No	0
None	0.5	none	No	0
Less than 50%	2	none	No	0
Less than 50%	3	none	No	0
More than 50%	2.5	none	No	0
Less than 50%	3	none	No	0
None	0	none	No	0
None	3.5	none	No	0
None	0	none	No	0
Less than 50%	2.5	none	Yes	0
None	0	none	No	0
Less than 50%	0	none	Yes	0
None	2	none	No	yes
None	1.5	none	No	0
Less than 50%	4	none	Yes	0
Less than 50%	2.1	none	No	0

Less than 50%	2.6	none	No	0
More than 50%	0	none	No	yes
Less than 50%	4	none	No	0
None	0.9	none	No	0
Less than 50%	6	none	No	0
None	1.8	none	No	0
Less than 50%	1	none	No	0
Less than 50%	1	none	No	0
None	0.4	none	No	0
None	0	none	No	0
Less than 50%	2.5	none	Yes	0
Less than 50%	2	none	No	0
Less than 50%	2.5	none	No	0
Less than 50%	1.3	none	No	0
None	0	none	No	0
None	0.8	none	No	0
Less than 50%	5	none	No	0
Less than 50%	2.9	none	No	0
None	0	none	No	0
Less than 50%	0.8	none	No	0
None	1	none	No	0
